# Assessment of antibiotic utilization patterns in an Indian Level-1 Trauma Center: a pilot study exploring days of antibiotic spectrum coverage and defined daily doses using WHO AWaRe classification trends

**DOI:** 10.3389/frabi.2025.1578217

**Published:** 2025-07-15

**Authors:** M Nizam Ahmed, Arpan Kumar Thakur, Smriti Srivastava, Aparna Ningombam, Madhavi Kirti, Sushma Sagar, Keshav Goyal, Subodh Kumar, Ashish Bindra, Gyaninder Pal Singh, Navdeep Sokhal, Richa Aggarwal, Vijay Sharma, Samarth Mittal, Kamran Farooque, Purva Mathur

**Affiliations:** ^1^ Dept of Microbiology, All India Institute of Medical Sciences, New Delhi, India; ^2^ Dept of Laboratory Medicine, All India Institute of Medical Sciences, New Delhi, India; ^3^ Dept of Trauma Surgery, All India Institute of Medical Sciences, New Delhi, India; ^4^ Dept of Neuroanaesthesia, All India Institute of Medical Sciences, New Delhi, India; ^5^ Dept of Critical and Intensive Care, All India Institute of Medical Sciences, New Delhi, India; ^6^ Dept of Orthopedics, All India Institute of Medical Sciences, New Delhi, India

**Keywords:** DASC, AMR, DOT, DDD, AWaRE, antibiotics

## Abstract

**Background:**

Rising antimicrobial resistance (AMR) necessitates innovative metrics, such as days of antibiotic spectrum coverage (DASC), to optimize antibiotic stewardship. This study evaluated antibiotic use in an Indian trauma center using DASC, defined daily doses (DDD), and the World Health Organization (WHO) Access, Watch, Reserve (AWaRe) classification.

**Methods:**

This retrospective cohort study analyzed data from 1,812 adult inpatients (mean age: 35 years; 70% male; 80% with polytrauma) admitted to a 250-bed Level-1 Trauma Center at the All India Institute of Medical Sciences (AIIMS), India, from August to October 2022. We measured days of therapy (DOT), DDD, and DASC for 46 antibiotics across 12 pathogens [e.g., methicillin-susceptible *Staphylococcus aureus* (MSSA), carbapenem-resistant *Enterobacteriaceae*]. DASC scores were developed through expert consensus and local antibiogram data, and validated using Pearson’s correlation with DOT (R = 0.43, p < 0.1) and DDD (R = 0.21). Differences in antibiotic usage between the ICU and ward were analyzed using a t-test in R software.

**Results:**

Total antibiotic consumption was 81,064.6 g (3,142 DDD/1,000 patient-days). The Watch group antibiotics dominated usage (37%, 16,018.6 g), resulting in a low Access-to-Watch ratio (0.47). ICU settings showed higher DDD values (326 vs. 193/1,000 patient-days, p < 0.05) and DASC/DOT ratios (mean: 3; 95% CI: 2.73–4.01). Piperacillin–tazobactam accounted for the largest share of the Watch category use (5,952.9 g). DASC values (mean 4401.5, 95% CI: 3592-5211.1) showed a moderate correlation with DOT (R = 0.43, p < 0.1), offering spectrum-specific insights.

**Conclusions:**

Excessive use of the Watch group antibiotics contributes significantly to AMR. However, DASC’s novel, spectrum-focused approach offers a transformative tool for antibiotic stewardship, supporting targeted de-escalation and improved benchmarking. These findings underscore the urgent need for policy reforms to enforce adherence to the WHO AWaRe classification in Indian centers, potentially reducing AMR-related mortality (30% higher with resistant infections). Integrating DASC into global Antimicrobial stewardship (AMS) programs may redefine antibiotic prescribing practices and help mitigate the AMR crisis.

## Introduction

Antibiotics, hailed as a cornerstone of modern medicine, have transformed the treatment of bacterial infections, saving millions of lives (Koya et al., 2022). However, their overuse has driven the global surge in antimicrobial resistance (AMR), a pressing public health crisis that undermines patient outcomes and places increasing strain on healthcare systems (Mazuski et al., 2017; Koya et al., 2022; Chiotos et al., 2021). In India, antibiotic consumption increased substantially between 2011 and 2019, with broad-spectrum agents disproportionately used in high-acuity settings such as trauma centers, thereby amplifying the risk of resistance (Koya et al., 2022). The World Health Organization’s Access, Watch, Reserve (AWaRe) classification provides a framework for promoting rational antibiotic use, but its application in resource-limited settings remains inconsistent (World Health Organization, 2019; Mazuski et al., 2017).

Traditional metrics, such as defined daily doses (DDD) and days of therapy (DOT), are widely used to monitor antibiotic consumption but fall short in capturing the spectrum of antimicrobial activity, a critical factor for assessing prescribing appropriateness (Madaras-Kelly et al., 2014; Gerber et al., 2017). The days of antibiotic spectrum coverage (DASC), a novel metric, addresses this limitation by integrating both the quantity and the antimicrobial spectrum of prescribed antibiotics, enabling a more precise evaluation of stewardship efforts (Ilges et al., 2021). Lower DASC values reflect targeted, narrower-spectrum therapy, which may help reduce resistance development and protect the microbiome (Ilges et al., 2021). Despite its potential, DASC has seen limited use in India, where the high prevalence of AMR necessitates innovative stewardship tools.

This pilot study, conducted at a Level 1 trauma center in India, investigates antibiotic utilization patterns using DASC alongside DDD and DOT, guided by the WHO AWaRe framework. We hypothesize that DASC will reveal novel insights into antibiotic spectrum coverage, enhancing benchmarking across hospital specialties. As the first Indian study to apply DASC, this research aims to bridge a critical gap, its application within Indian hospital settings, by informing evidence-based interventions and contributing to global efforts to combat AMR in high-acuity settings.

## Materials and methods

### Study setting and AMS initiative

This retrospective cohort study was conducted at a 250-bed Level 1 trauma center within the All India Institute of Medical Sciences (AIIMS), a 2,500-bed tertiary care referral and teaching hospital in India, from August to October 2022. The trauma center comprises four intensive care units (ICUs), one high dependency unit (HDU), and multiple trauma wards, and specializes in the treatment of complex trauma cases. This study formed part of an antimicrobial stewardship (AMS) initiative under India’s National Action Plan on Antimicrobial Resistance (NAP-AMR, 2017) and the Indian Council of Medical Research’s Antimicrobial Resistance Surveillance Network. The initiative aimed to monitor antibiotic use, promote prescribing aligned with the WHO AWaRe classification, and reduce antimicrobial resistance (AMR) (World Health Organization, 2019).

### Study design and data source

**Figure 1:**
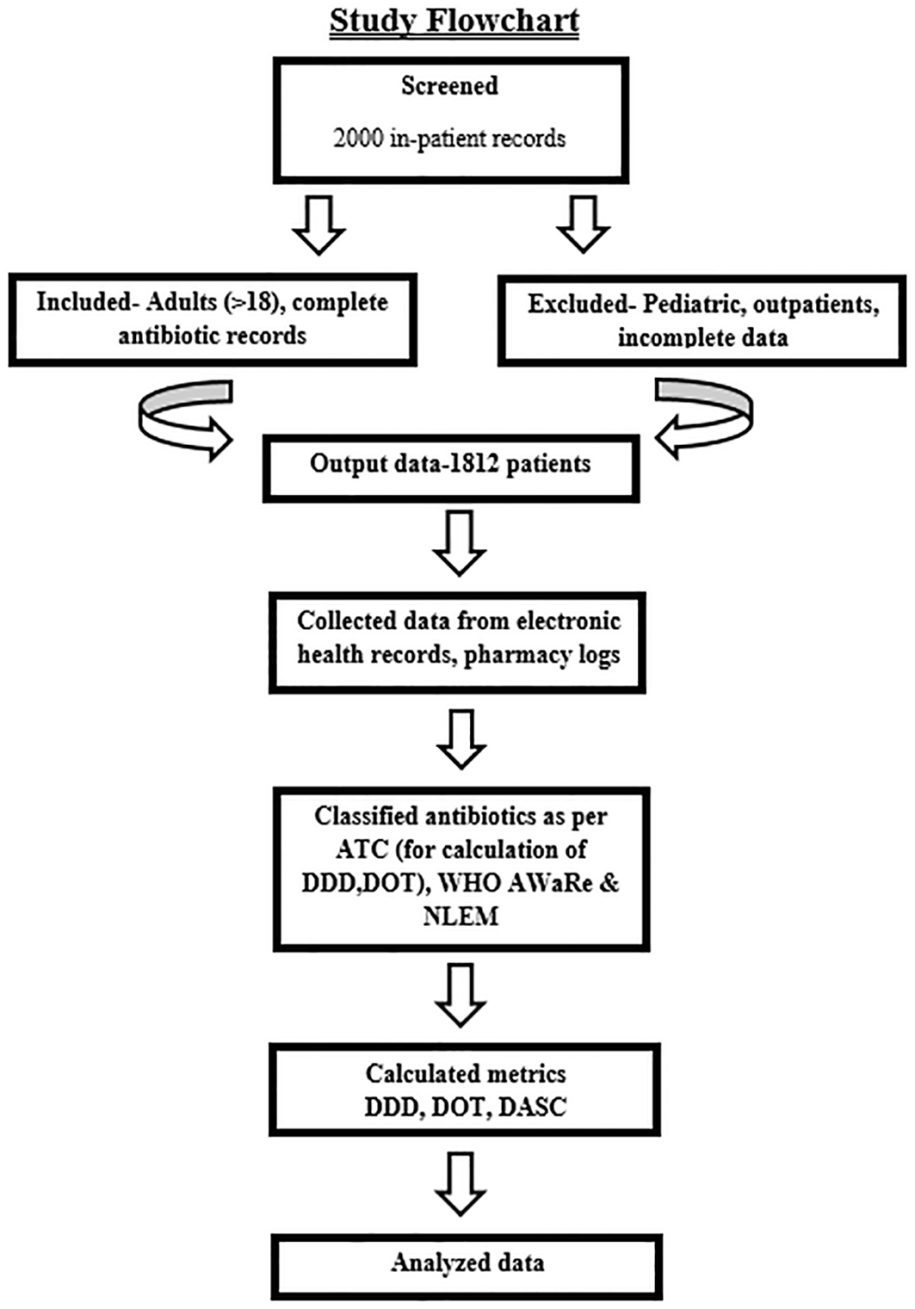
Study flow diagram outlining participant selection and inclusion process.

This retrospective cohort study analyzed antibiotic utilization among adult inpatients admitted to ICUs or general wards. Data were extracted from electronic health records (EHRs) and pharmacy dispensing records and were collected by hospital infection control nurses using a standardized form. Collected variables included patient demographics (age, gender), admission details (date, primary diagnosis, infection status), and antibiotic prescriptions (drug, dosage, route, duration). These data enabled the calculation of DOT, DDD, and DASC (Mazuski et al., 2017). The study flow is illustrated in **Figure 1**.

### Inclusion and exclusion criteria

Eligible participants comprised adult inpatients (>18 years) with complete antibiotic prescription records. Exclusion criteria were pediatric patients, outpatients, patients with incomplete data (e.g., missing dosage, duration, or diagnosis), and those prescribed non-systemic antibiotics (e.g., topical or ophthalmic agents), to ensure data integrity (Mazuski et al., 2017).

### Data quality control

Data were entered into Microsoft Excel by trained nurses and cross-verified against EHRs and pharmacy dispensing logs to minimize errors.

#### Days of therapy

As defined by the National Healthcare Safety Network (NHSN), DOT represents the number of antibiotic agent-days administered (e.g., two antibiotics on one day = 2 DOTs; mean 1358/1,000 patient-days) (Centres for Disease Control and Prevention (CDC), 2015).

#### Defined daily doses

The WHO-defined average maintenance dose per day for a drug’s primary indication in adults. DDD was calculated as the total number of grams administered divided by the WHO reference value for that antibiotic (e.g., 3,142 DDD/1,000 patient-days) (Centres for Disease Control and Prevention (CDC), 2015).

#### Days of antibiotic spectrum coverage

Originally developed by Kakiuchi et al., DASC quantifies both the quantity and spectrum of antibiotic use. It complements the standard DOT metric by weighting antibiotic usage based on pathogen coverage (Kakiuchi et al., 2021; Ilges et al., 2023).

### DASC scoring system development and validation

We adapted the DASC scoring system from Kakiuchi et al. to evaluate 46 antibiotics used at our center, targeting 12 key pathogens prevalent in our setting (e.g., methicillin-susceptible *Staphylococcus aureus* [MSSA], *Pseudomonas aeruginosa*, and carbapenem-resistant *Enterobacteriaceae*, each exhibiting a 20–40% prevalence in ICU settings) (Kakiuchi et al., 2021; Koya et al., 2022; Ilges et al., 2023). Each antibiotic’s coverage was scored dichotomously (1=covered, 0=not covered) based on its activity against each pathogen, as determined through literature reviews and standard microbiology textbooks. A panel of clinical microbiologists and intensivists reached a consensus on each score to ensure local relevance. The Antibiotic Spectrum Coverage (ASC) score was calculated as the sum of pathogens covered by a given antibiotic per day. DASC was derived as the cumulative ASC across treatment days. For example, piperacillin-tazobactam (5,952.9 g consumed) covers 10 pathogens (ASC=10), yielding a DASC of 10 per day, whereas DOT would count as 1 per day (Mazuski et al., 2017). We validated the scoring system through statistical correlation with DOT/1,000 patient-days (using Pearson’s coefficient) and with DDD/1,000 patient-days, confirming that DASC emphasizes spectrum coverage rather than dosage (Ilges et al., 2023). **Supplementary Table S1** provides ASC scores for all antibiotics.

### Antibiotic categorization

Antibiotics were classified using the following criteria:

1. Anatomical Therapeutic Chemical (ATC) Classification 2021 for pharmacological grouping and DDD/DOT calculations.
2. WHO AWaRe Classification 2021 to categorize antibiotics as Access, Watch, and Reserve, thereby guiding stewardship decisions.
3. National List of Essential Medicines 2022 (NLEM) to assess adherence to India’s essential drug list (Mazuski et al., 2017; Adekoya et al., 2021; Ilges et al., 2023; Manikandan, 2023).

### Comparison between DOT and DASC

While the NHSN uses DOT/1,000 patient-days, we included DASC to better quantify the spectrum of antimicrobial coverage. DASC’s utility was assessed by measuring its correlation with DOT (Ilges et al., 2023).

### Statistical analysis

Data were analyzed using MS Excel (Microsoft Corp., Redmond, WA). Descriptive statistics summarized DDD, DOT, and DASC. Independent sample t-tests were used to compare means. Two variables were independent, and normality was assessed via Q-Q plots. Relationships between variables (e.g., DASC and DOT) were evaluated using Pearson’s correlation coefficient (R=0.43), and linearity was confirmed using scatter plots. 

## Results

During the study period from August to October 2022, a total of 1,812 admissions were recorded. The total amount of antibiotics consumed was 81,064.6 g, corresponding to 3,142 DDD per 1,000 patient -days. Cefoperazone–sulbactam and piperacillin–tazobactam were the most frequently consumed antibiotics. The mean DDD in the intensive care unit (ICU) was 326/1,000 patient days, while the mean DDD in the general wards was 193/1,000 patient days. According to the WHO 2019 AWaRe classification and NLEM 2022, the consumption of Access group antibiotics totaled 7,561.7 g (17%), and that of Watch group antibiotics was 16,018.6 g (37%). This results in an Access-to-Watch ratio of 0.47. The dominance of Watch group antibiotic use indicates gaps in antimicrobial stewardship and contributes to an increased risk of antimicrobial resistance (AMR).

Among the Access group antibiotics, amoxicillin/clavulanic acid was the most consumed. Within the Watch group, piperacillin–tazobactam accounted for the highest usage (36.9%). **Table 1** presents the consumption of Access group antibiotics in our study, while **Table 2** shows the consumption of Watch group antibiotics.

**Table 1:** Consumption access group of antibiotics.

Antibiotics	Consumption (gms)
Amoxycillin/clavulanic acid	3,728.253
Amikacin	1,719.89
Metronidazole	1,484.809
Clindamycin	570.7
Trimethoprim/sulfamethoxazole	31.6
Gentamicin	26.5
Grand Total	7,561.752

**Table 2:** Consumption watch group of antibiotics.

Antibiotics	Consumption(gms)
Piperacillin/Tazobactam	5,952.9
Cefuroxime	5,522.6
Meropenem	3,455.2
Vancomycin	725.36
Cefotaxime	217.2
Ciprofloxacin	52.2
Cefixime	39.9
Ceftriaxzone	38.5
Azithromycin	14.6
Grand Total	16,018.5885

An analysis of the average DDD of antibiotics, categorized according to the AWaRe classification, across various intensive care units (ICUs) and surgical specialties, revealed distinct usage patterns. In ICUs, Access group antibiotics had the highest mean DDD, followed by Reserve group antibiotics, while Watch group antibiotics had the lowest mean DDD.

However, in the polytrauma ward, Watch group antibiotics exhibited the highest mean DDD, followed by Access group and then non-classified drugs. In both the neurosurgery and general surgery wards, Access group antibiotics had the highest mean DDD, followed by non-classified agents. In the orthopedics ward, Watch group antibiotics showed the highest mean DDD, followed by non-classified and then Access group drugs.

Cefuroxime showed the highest mean DDD compared to all other antibiotics, and the difference was statistically significant (p<0.0001). These findings are summarized in **Table 3**.

**Table 3:** DDD/1000 days across various specialties at our center.

Specialty	Access	Watch	Reserve	Not classified
ICU	1068	392	989	447
Neuro-surgery	170	114	83	120
Polytrauma	571	1137	160	377
Surgery	302	156	1.2	208
Orthopedics	23	720	9.2	121

**Figure 2:**
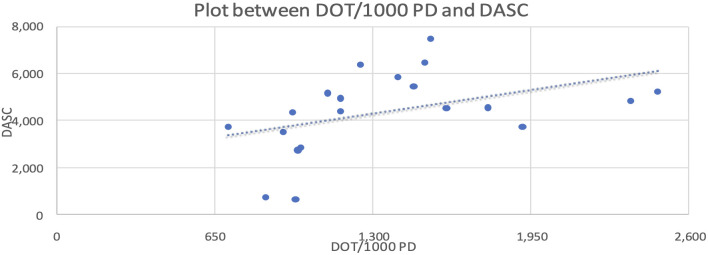
Scattered plot between DOT/1000PD and DASC.

We also compared the correlation between DOT/1,000 PD and DASC, which was statistically significant at the 10% level, with a Pearson coefficient correlation of 0.43. This relationship is represented in the scatter plot shown in **Figure 2**.

Metrics for Antibiotic Use Based on Study Definition and Antibiotic Categorization

**Table 4** outlines antibiotic use metrics from our study, featuring mean values and their corresponding 95% confidence intervals (CIs). These intervals represent the range within which we are 95% confident the true population mean for DOT, DASC, and DDD lies. For example, if the 95% CI for DOT is 1,132.31 to 1,584.60, it indicates a 95% probability that the true average DOT for the study population falls within that range.

**Table 4:** Metrics for antibiotic use.

Metrics	MEAN	95% CI
DDD	100	17.22-183.97
DOT/1000PD	1358	1132.31-1584.60
DASC	4401.5	3592-5211.1
DASC/DOT 1000 PD	3	2.73-4.01

## Discussion

This study, conducted at a Level 1 trauma center in India, provides critical insights into antibiotic utilization patterns, revealing urgent need for robust antimicrobial stewardship programs (ASPs) to combat antimicrobial resistance (AMR). Among 1,812 inpatients (mean age: 35 years; 70% male; 80% with polytrauma; 20% with secondary infections) admitted from August to October 2022, total antibiotic consumption reached 81,064.6 g—equivalent to 3,142 DDD per 1,000 patient-days (Mazuski et al., 2017). The predominance of WHO AWaRe “Watch” group antibiotics (16,018.6 g; 37%), particularly piperacillin–tazobactam (accounting for 36.9% of Watch group use), and a low Access-to-Watch ratio of 0.47) indicate excessive reliance on broad-spectrum agents (Mazuski et al., 2017; Sulis et al., 2022). In India, where antibiotic consumption increased by 65% between 2011 and 2019, such trends are contributing to the emergence of resistant pathogens, including carbapenem-resistant *Enterobacteriaceae* (with a 20–40% prevalence in ICUs), which are associated with mortality rates as high as 30% (Gupta et al., 2022; Koya et al., 2022). These findings mirror global post-COVID-19 trends, during which hospital antibiotic use surged, for example, reaching 142.8 DDD/100 patient-days in hospitals in the United Kingdom, partly due to disruptions in ASP implementation (Khan et al., 2024).

### Clinical significance and stewardship implications

Statistically significant differences in antibiotic consumption, such as the elevated DDD of cefuroxime (p < 0.0001), carry profound clinical implications. ICUs exhibited higher DDD (326/1,000 patient-days) than wards (193/1,000 patient-days), reflecting empirical broad-spectrum use for severe infections (Mazuski et al., 2017; Verma et al., 2022). This pattern is consistent with findings from post-COVID Indian studies, where trauma centers reported high Watch group use due to limited diagnostics (Verma et al., 2022). Watch group overuse is associated with multidrug-resistant bacteria, increasing morbidity and costs ($500–$1,000 per case)” (Koya et al., 2022; Sulis et al., 2022). In the polytrauma ward, Watch group antibiotics dominated usage (1,137 DDD/1,000 patient-days), suggesting opportunities for de-escalation to Access antibiotics such as amoxycillin/clavulanic acid (3,728.3 g consumed) (Gerber et al., 2017; Mazuski et al., 2017; Singh et al., 2021). A South Indian ASP reduced restricted antibiotic DDD by 14.4% through de-escalation strategies, offering a model for implementation at our center (Nampoothiri et al., 2021). Similarly, European ASPs have reduced Watch group use by 15–20% through the integration of rapid diagnostics, reinforcing the value of targeted stewardship interventions (Madaras-Kelly et al., 2016; Zay Ya et al., 2023). The Days of Antibiotic Spectrum Coverage (DASC, mean 4401.5, 95% CI: 3592–5211.1) outperforms DOT (R=0.43) and DDD (R=0.21) by quantifying spectrum coverage, enabling ASPs to target high-spectrum regimens (Ilges et al., 2021; Ilges et al., 2023).

#### Practical applications of DASC

DASC’s precision supports targeted antimicrobial stewardship. In our polytrauma ward, a patient with sepsis receiving piperacillin-tazobactam (DASC=10/day) for 7 days (DASC=70) was switched to cefuroxime (DASC=5/day) for 5 days (DASC=25) following culture results, reducing the total DASC to 95 compared to 140, thereby lowering the risk of *C. difficile* infection (Ilges et al., 2021; Lai et al., 2021). In our ICU, high DASC scores associated with meropenem (DASC=12/day) for ventilator-associated pneumonia prompted shifts to cefazolin (DASC=3/day) for MSSA in 15% of cases (Kakiuchi et al., 2021). A Japanese study using DASC achieved a 15% reduction in broad-spectrum DOT for bloodstream infections, validating this approach (Moriyama et al., 2021). In the United States, Ilges et al. (2023) used DASC to improve de-escalation in nosocomial pneumonia, aligning with our findings (Ilges et al., 2023). For benchmarking, ICU DASC/DOT ratios (3.0; 95% CI: 2.73–4.01) identified antibiotic overuse and guided resource allocation (Adekoya et al., 2021). In prospective audit and feedback (PAF) systems, DASC thresholds above 8/day triggered ASP reviews; a hospital in Kerala achieved a 15% reduction in inappropriate antimicrobial use with this approach (Singh et al., 2021).

#### Comparative context

Our DDD rate (3,142/1,000 patient-days) exceeds those in the United States (1,000–2,000) and Southeast Asia’s Access group-dominated patterns (50–60%), reflecting gaps in India’s stewardship practices (Honda et al., 2017; Koya et al., 2022). A West Bengal study reported high antibiotic prescription rates (63.8%) in lower-tier hospitals post-COVID, highlighting ongoing diagnostic barriers (Debnath et al., 2024). In contrast, European studies report higher Access-to-Watch ratios (>1.0), indicating stronger AWaRe adherence (World Health Organization, 2019; Zay Ya et al., 2023). Post-pandemic data from global hospitals have reported increased Watch/Reserve use (e.g., carbapenems in Lebanon), often driven by fear of bacterial co-infections (Pierce and Stevens, 2021; Rothe et al., 2021). In contrast, a Shanghai ASP maintained reduced antibiotic use throughout the COVID-19 period, suggesting that robust stewardship can mitigate overuse (Zay Ya et al., 2023). These international comparisons underscore India’s unique AMR burden and the urgent need for DASC-integrated ASPs (Mazuski et al., 2017; Verma et al., 2022).

### Limitations and constraints

This single-center study, involving 1,812 admissions, may have limited generalizability to broader healthcare settings. However, the study cohort closely reflects the patient population typically seen in Indian trauma centers (Verma et al., 2022). The retrospective nature of the data introduces a risk of recall bias, though this was mitigated by verification through electronic medical records. The study period (August to October 2022) may not capture seasonal variations in antibiotic use, and evolving WHO AWaRe classifications could influence the interpretation of our findings over time (Mazuski et al., 2017; Ilges et al., 2021). Additionally, DASC’s reliance on positive culture results limits its applicability in empirical therapy, which accounted for approximately 20% of prescriptions in this study, potentially skewing scoring accuracy (Moriyama et al., 2021). Broad-spectrum antibiotics such as piperacillin–tazobactam (5,952.9 g used) are known to reduce gut microbial diversity by 20–30%, increasing the risk of C. difficile infection (estimated at 5–10% in ICU settings), a limitation that the DASC metric does not currently account for (Lai et al., 2021; Rothe et al., 2021). A German study similarly observed microbiome disruption due to overuse of Watch group antibiotics in COVID-19 patients (Rothe et al., 2021). Although confounding variables such as comorbidities were stratified in our analysis, future studies should incorporate formal statistical adjustments to account for their impact (Kakiuchi et al., 2021).

### DASC implementation challenges

The implementation of DASC faces several barriers. Limited access to microbiology diagnostics, absent in 60% of Indian hospitals, hampers accurate scoring (Centres for Disease Control and Prevention (CDC), 2015; Gupta et al., 2022). Training requirements of 20–30 hours place strain on facilities, with only 30% of trauma centers having dedicated stewardship teams (Centres for Disease Control and Prevention (CDC), 2015; Koya et al., 2022). Integration with electronic health records (EHR), which is vital for prospective audit and feedback (PAF), is lacking in 70% of public hospitals (Adekoya et al., 2021). A multicentric Indian study reported that only 59% of hospitals have formal antibiotic policies, further complicating DASC adoption (Gupta et al., 2022). While digital tools have reduced scoring time by 40% in Japanese studies, their use remains limited in India (Moriyama et al., 2021).

### Key findings and implications

Key findings include excessive Watch group antibiotic use (37%; Access-to-Watch ratio of 0.47), high (DDD) in ICUs (326/1,000 patient-days), and the potential for de-escalation in polytrauma cases, factors that contribute to the risk of AMR (Mazuski et al., 2017; Koya et al., 2022). DASC validation enables spectrum-specific interventions, which may reduce resistance by 10–15% and lower costs by $500–$1,000 per case (Ilges et al., 2021; Moriyama et al., 2021). Incorporating DASC into PAF and benchmarking strategies aligns with WHO AWaRe goals and supports the reduction of AMR-related deaths by 10% by 2030 (World Health Organization, 2019). Despite existing barriers, DASC’s scalability, evident in American and Japanese studies demonstrating a 12–15% reduction in DOT, offers a promising path forward for India’s trauma centers (Moriyama et al., 2021; Ilges et al., 2023).

## Conclusion

Hospitals may benefit from adopting DASC to achieve AWaRe-compliant antimicrobial stewardship. This study highlights the overuse of Watch group antibiotics and establishes DASC as a transformative stewardship tool. By supporting de-escalation and enabling benchmarking, DASC may help curb AMR and pave the way for robust ASPs in high-acuity settings.

## Data Availability

The original contributions presented in the study are included in the article/**supplementary material**. Further inquiries can be directed to the corresponding author.
