# Supplementary material for: Assessment of antibiotic utilization patterns in an Indian Level-1 Trauma Center: a pilot study exploring days of antibiotic spectrum coverage and defined daily doses using WHO AWaRe classification trends
Source: Front Antibiot. 2025 Jul 15;4:1578217. doi: 10.3389/frabi.2025.1578217 (PMC12304517; doi:10.3389/frabi.2025.1578217)
Supplement: Supplementary file 1 [file DataSheet1.docx]

**Supplementary material
Table (Supplementary) - Calculation for Antibiotic Spectrum Coverage (ASC) score**

| **Antibiotic Class** | **Antibiotics** | **Total ASC** | **Staphylococcus aureus** | **Streptococcus** | **Enterococcus** | **Anaerobes** | **E. coli/Klebsiella pneumoniae** | **Enterobacter/Serratia/**  **Citrobacter** | **Pseudomonas aeruginosa** | **Acinetobacter baumannii** | **Atypical** | **MRSA** | **VRE** | **CRE** |
| --- | --- | --- | --- | --- | --- | --- | --- | --- | --- | --- | --- | --- | --- | --- |
| **Aminoglycoside** | Amikacin | 6 | 1 | 0 | 0 | 0 | 1 | 1 | 1 | 1 | 0 | 1 | 0 | 0 |
|  | Gentamicin | 6 | 1 | 0 | 0 | 0 | 1 | 1 | 1 | 1 | 0 | 1 | 0 | 0 |
|  | Tobramycin | 7 | 1 | 0 | 1 | 0 | 1 | 1 | 1 | 1 | 0 | 0 | 0 | 0 |
| **β-lactam/β-lactamase inhibitor**  **(BLBLI)** | Amoxicillin/clavulanate | 5 | 1 | 1 | 1 | 1 | 1 | 0 | 0 | 0 | 0 | 0 | 0 | 0 |
|  | Ampicillin-Sulbactam | 5 | 1 | 1 | 0 | 1 | 1 | 0 | 0 | 1 | 0 | 0 | 0 | 0 |
|  | Ceftazidime-Avibactam | 6 | 0 | 1 | 0 | 0 | 1 | 1 | 1 | 1 | 0 | 0 | 0 | 1 |
|  | Ticarcillin-Clavulanate | 7 | 1 | 1 | 0 | 1 | 1 | 1 | 1 | 1 | 0 | 0 | 0 | 0 |
|  | Piperacillin tazobactam | 8 | 1 | 1 | 1 | 1 | 1 | 1 | 1 | 1 | 0 | 0 | 0 | 0 |
| **Carbapenems** | Ertapenem | 5 | 1 | 1 | 0 | 1 | 1 | 0 | 0 | 0 | 0 | 1 | 0 | 0 |
|  | Doripenem | 7 | 1 | 1 | 0 | 1 | 1 | 1 | 1 | 1 | 0 | 0 | 0 | 0 |
|  | Imipenem | 7 | 1 | 1 | 0 | 1 | 1 | 1 | 1 | 1 | 0 | 0 | 0 | 0 |
|  | Meropenem | 7 | 1 | 1 | 0 | 1 | 1 | 1 | 1 | 1 | 0 | 0 | 0 | 0 |
| **Cephalosporin** | Cefixime | 2 | 0 | 1 | 0 | 0 | 1 | 0 | 0 | 0 | 0 | 0 | 0 | 0 |
|  | Cefuroxime | 3 | 1 | 1 | 0 | 0 | 1 | 0 | 0 | 0 | 0 | 0 | 0 | 0 |
|  | Cefotaxime | 3 | 1 | 1 | 0 | 0 | 1 | 0 | 0 | 0 | 0 | 0 | 0 | 0 |
|  | Cefoxitin | 4 | 1 | 1 | 0 | 1 | 1 | 0 | 0 | 0 | 0 | 0 | 0 | 0 |
|  | Ceftazidime | 5 | 0 | 1 | 0 | 0 | 1 | 1 | 1 | 1 | 0 | 0 | 0 | 0 |
|  | Ceftriaxone | 4 | 1 | 1 | 0 | 0 | 1 | 1 | 0 | 0 | 0 | 0 | 0 | 0 |
|  | Cefepime | 6 | 1 | 1 | 0 | 0 | 1 | 1 | 1 | 1 | 0 | 0 | 0 | 0 |
| **Fluoroquinolones** | Ciprofloxacin | 9 | 1 | 1 | 0 | 1 | 1 | 1 | 1 | 1 | 1 | 1 | 0 | 0 |
|  | Norfloxacin | 7 | 1 | 0 | 0 | 0 | 1 | 1 | 1 | 1 | 1 | 1 | 0 | 0 |
|  | Levofloxacin | 9 | 1 | 1 | 1 | 0 | 1 | 1 | 1 | 1 | 1 | 1 | 0 | 0 |
| **Folate pathway inhibitor** | Trimethoprim/sulfamethoxazole | 6 | 1 | 1 | 0 | 0 | 1 | 1 | 0 | 1 | 0 | 1 | 0 | 0 |
| **Fosfomycin** | Fosfomycin | 8 | 1 | 1 | 1 | 0 | 1 | 1 | 0 | 0 | 0 | 1 | 1 | 1 |
| **Glycopeptide** | Vancomycin | 4 | 1 | 1 | 1 | 0 | 0 | 0 | 0 | 0 | 0 | 1 | 0 | 0 |
|  | Teicoplanin | 4 | 1 | 1 | 1 | 0 | 0 | 0 | 0 | 0 | 0 | 1 | 0 | 0 |
| **Glycylcyline** | Tigecycline | 11 | 1 | 1 | 1 | 1 | 1 | 1 | 0 | 1 | 1 | 1 | 1 | 1 |
| **Lincosamide** | Clindamycin | 4 | 1 | 1 | 0 | 1 | 0 | 0 | 0 | 0 | 0 | 1 | 0 | 0 |
| **Lipopeptide** | Daptomycin | 5 | 1 | 1 | 1 | 0 | 0 | 0 | 0 | 0 | 0 | 1 | 1 | 0 |
| **Macrolide** | Erythromycin | 4 | 1 | 1 | 0 | 0 | 0 | 0 | 0 | 0 | 1 | 1 | 0 | 0 |
|  | Azithromycin | 6 | 1 | 1 | 0 | 1 | 1 | 0 | 1 | 0 | 1 | 0 | 0 | 0 |
| **Monobactam** | Aztreonam | 3 | 0 | 0 | 0 | 0 | 1 | 1 | 1 | 0 | 0 | 0 | 0 | 0 |
| **Nitrofuran** | Nitrofurantoin | 5 | 1 | 1 | 1 | 0 | 1 | 0 | 0 | 0 | 0 | 0 | 1 | 0 |
| **Nitroimidazole** | Metronidazole | 2 | 0 | 0 | 0 | 1 | 0 | 0 | 0 | 0 | 0 | 1 | 0 | 0 |
| **Oxazolidinone** | Linezolid | 4 | 1 | 1 | 1 | 0 | 0 | 0 | 0 | 0 | 0 | 0 | 1 | 0 |
| **Penicillin** | Penicillin G,V | 3 | 0 | 1 | 1 | 1 | 0 | 0 | 0 | 0 | 0 | 0 | 0 | 0 |
|  | Amoxicillin | 4 | 0 | 1 | 1 | 1 | 1 | 0 | 0 | 0 | 0 | 0 | 0 | 0 |
|  | Ampicillin | 4 | 0 | 1 | 1 | 1 | 1 | 0 | 0 | 0 | 0 | 0 | 0 | 0 |
| **Phenicol** | Chloramphenicol | 7 | 1 | 1 | 1 | 1 | 1 | 0 | 0 | 0 | 0 | 1 | 1 | 0 |
| **Polymyxin** | Colistin | 4 | 0 | 0 | 0 | 0 | 1 | 0 | 1 | 1 | 0 | 0 | 0 | 1 |
|  | Polymyxin-B | 4 | 0 | 0 | 0 | 0 | 1 | 0 | 1 | 1 | 0 | 0 | 0 | 1 |
| **Rifampin** | Rifampicin | 3 | 1 | 1 | 0 | 0 | 0 | 0 | 0 | 0 | 1 | 0 | 0 | 0 |
| **Tetracycline** | Tetracycline | 4 | 1 | 1 | 0 | 0 | 1 | 0 | 0 | 0 | 1 | 1 | 0 | 0 |
|  | Minocycline | 5 | 1 | 1 | 0 | 0 | 1 | 0 | 0 | 1 | 1 | 1 | 0 | 0 |
